# Design, Recruitment, and Baseline Characteristics of a Virtual 1-Year Mental Health Study on Behavioral Data and Health Outcomes: Observational Study

**DOI:** 10.2196/17075

**Published:** 2020-07-23

**Authors:** Shefali Kumar, Jennifer L A Tran, Ernesto Ramirez, Wei-Nchih Lee, Luca Foschini, Jessie L Juusola

**Affiliations:** 1 Evidation Health San Mateo, CA United States

**Keywords:** mental health, anxiety, depression, behavioral data

## Abstract

**Background:**

Depression and anxiety greatly impact daily behaviors, such as sleep and activity levels. With the increasing use of activity tracking wearables among the general population, there has been a growing interest in how data collected from these devices can be used to further understand the severity and progression of mental health conditions.

**Objective:**

This virtual 1-year observational study was designed with the objective of creating a longitudinal data set combining self-reported health outcomes, health care utilization, and quality of life data with activity tracker and app-based behavioral data for individuals with depression and anxiety. We provide an overview of the study design, report on baseline health and behavioral characteristics of the study population, and provide initial insights into how behavioral characteristics differ between groups of individuals with varying levels of disease severity.

**Methods:**

Individuals who were existing members of an online health community (Achievement, Evidation Health Inc) and were 18 years or older who had self-reported a diagnosis of depression or anxiety were eligible to enroll in this virtual 1-year study. Participants agreed to connect wearable activity trackers that captured data related to physical activity and sleep behavior. Mental health outcomes such as the Patient Health Questionnaire (PHQ-9), the Generalized Anxiety Disorder Questionnaire (GAD-7), mental health hospitalizations, and medication use were captured with surveys completed at baseline and months 3, 6, 9, and 12. In this analysis, we report on baseline characteristics of the sample, including mental health disease severity and health care utilization. Additionally, we explore the relationship between passively collected behavioral data and baseline mental health status and health care utilization.

**Results:**

Of the 1304 participants enrolled in the study, 1277 individuals completed the baseline survey and 1068 individuals had sufficient activity tracker data. Mean age was 33 (SD 9) years, and the majority of the study population was female (77.2%, 994/1288) and identified as Caucasian (88.3%, 1137/1288). At baseline, 94.8% (1211/1277) of study participants reported experiencing depression or anxiety symptoms in the last year. This baseline analysis found that some passively tracked behavioral traits are associated with more severe forms of anxiety or depression. Individuals with depressive symptoms were less active than those with minimal depressive symptoms. Severe forms of depression were also significantly associated with inconsistent sleep patterns and more disordered sleep.

**Conclusions:**

These initial findings suggest that longitudinal behavioral and health outcomes data may be useful for developing digital measures of health for mental health symptom severity and progression.

## Introduction

### Background

The increasing use of wearable devices capable of tracking behavioral activities like steps and sleep bring opportunities for a more nuanced understanding of the impact of mental health conditions on daily life. Conventional approaches to behavioral research in the form of periodic surveys or outreach cannot provide sufficient information on the day-to-day or real-world experiences of people with mental health conditions. However, current wearable activity devices can passively capture behavioral information with time granularities to the minute and even second level, and there is growing interest in how these data can be used and applied in research and health care settings [1]. Data from these devices are usually collected passively and continuously, and can provide unique longitudinal insights into an individual’s behavior (eg, sleep and activity patterns) that cannot be measured by traditional methods. Researchers have begun to leverage activity trackers, health and fitness apps, and other digital technologies in clinical and health outcome studies to collect behavioral data and analyze it alongside more conventional types of health outcomes and clinical data [2]. These joint data sets can then be used to identify various behavioral characteristics and patterns associated with different health conditions and can help provide a better understanding of disease status, onset, and progression, thus potentially leading to improved screening and monitoring techniques, therapeutic innovations, and disease management [3].

A therapeutic area of particular interest is mental health, specifically depression and anxiety, as these conditions impact an individual’s daily activities, behaviors, and health-related quality of life. Mental health illnesses affect nearly 20% of the adult population in the United States [4], and depression is forecasted to be the second leading cause of disability throughout the world in 2020 [5]. Mental health illnesses are a risk factor for many other chronic diseases and place a significant burden on the individual, their community, and the overall health care system [5]. Given that mental health illnesses can have a bidirectional relationship with health behaviors such as sleep and physical activity [6,7], it is a particularly appropriate therapeutic area to explore to further understand how passively collected behavioral data can be used to provide insight into an individual’s overall health outcomes.

### Prior Work

Previous studies have shown a correlation between self-reported physical activity levels and mental health illnesses, yet few studies have evaluated passively collected activity tracker–based data in individuals with mental health illnesses [8]. One small study analyzed self-reported health outcomes and smartphone sensor data and found correlations between changes in depression symptoms and speech patterns, geospatial activity, and sleep [8]. A recent larger study used tracking devices and heart rate monitors to understand how passively collected data correlated with a traditional patient-reported questionnaire, and found that increased physical activity was associated with better psychological well-being [9]. However, despite these initial signals, digital measures of mental health status, or digital signals and algorithms that can identify or characterize disease onset, severity, and progression, have yet to be precisely developed and validated [10].

### Goal of This Study

To better understand the relationship between behaviors and health outcomes for individuals with mental health conditions, and to begin to develop digital measures of mental health status, symptoms, and severity, we designed and launched a virtual 1-year prospective observational study where we collected self-reported health outcomes and utilization data alongside activity tracker–based behavioral data for individuals with depression or anxiety. This study has been fully recruited and data collection has been completed. The purpose of this manuscript is two-fold. First, this manuscript provides an overview of the study design, outlines the purely virtual operationalization of the protocol, and reports baseline health and behavioral characteristics of the study population. Second, this manuscript provides initial insights into how behavioral characteristics differ between groups of individuals with varying levels of disease severity.

## Methods

### Study Overview

This 1-year prospective observational study was designed with the objective of creating a novel longitudinal data set that combines self-reported health outcomes, health care utilization, and quality of life data with app- and activity tracker–based behavior data for individuals with depression and anxiety to support novel research exploring possible associations between longitudinal objective measures of health behaviors and self-reported mental health status.

This study was conducted completely virtually using a novel online study platform (Achievement Studies, Evidation Health Inc). Achievement is currently available in the United States with a research community of users that range in age from 18 to >80 years. Individuals have collectively self-reported on over 900,000 conditions. Achievement members can connect their activity trackers and fitness and health apps to the platform; as members log activities and use their activity trackers, they accumulate points that are redeemable for monetary rewards. Additionally, Achievement users are recruited for various studies based on the study criteria and can access Achievement on the web as well as through iPhone and Android apps. In this study, individuals were able to access the online study platform to complete study procedures and keep track of their progress throughout the study through the use of any web-enabled device. Participants were able to reach out to research staff with questions via email or phone before and during the enrollment process, and could continue to reach out throughout the study. This study was approved by the Solutions Institutional Review Board.

### Recruitment and Screening

We invited existing members of the Achievement online health community to participate in this study. Individuals who lived in the United States, were at least 18 years of age, and self-reported a diagnosis of depression or anxiety were eligible for the study. To assess their eligibility, potential participants completed a set of screener questions on the online study platform. If deemed eligible, they were then asked to sign an electronic informed consent form on the online study platform to enroll in the study.

### Data Collection

Once enrolled in the study, participants were asked to complete a baseline questionnaire, which consisted of questions about their health status, health care utilization, and treatment patterns. The baseline questionnaire also included questions from the Patient Health Questionnaire (PHQ-9) [11] and the Generalized Anxiety Disorder Questionnaire (GAD-7) [12] to detect and assess depression and anxiety severity levels, respectively.

Participants were then asked to connect at least one of the following activity trackers or fitness and health apps to their study dashboard: Fitbit, Withings (formerly Nokia Health), Garmin, Jawbone, Misfit, MyFitnessPal, or Apple Health. Since all participants were existing members of the online health community, most already had apps and trackers connected, but they were given the option to connect additional apps and trackers. Participants were not required to connect a tracker or app to continue in the study. As part of the consent process, participants were informed that the behavioral data included in the research analysis would consist of app- and tracker-based step, sleep, weight, and food logging data from the year prior to enrolling in the study and for the 1-year duration of the study. This data was collected passively through the study platform for the duration of the study.

At the 3-month, 6-month, 9-month, and 12-month (study end) time points of the study, participants were asked to complete online follow-up assessments. These assessments consisted of questions regarding the participant’s health status, health care utilization, and changes in treatment, and included the PHQ-9 and GAD-7 questionnaires.

### Measurements

In this study, the two validated patient-reported outcomes that we focus on are the 9-item PHQ-9 and the 7-item GAD-7, both of which can be self-administered. The PHQ-9 asks participants to rate on a scale of 0 (not at all) to 3 (nearly every day) the 9 DSM-IV criteria for depression. The GAD-7 asks individuals to assess the extent to which they have been bothered by various anxiety-related symptoms on a scale of 0 (not at all) to 3 (nearly every day).

To examine how tracker-based behavioral characteristics are associated with disease severity, we used four primary methods to classify participants based on the severity of their mental health condition(s). Methods 1 and 2 were based on thresholds of the two validated self-reported health outcomes questionnaires (PHQ-9 and GAD-7). In Method 1, we categorized participants based on their baseline PHQ-9 score; participants either had “depressive symptoms” (defined as having a PHQ-9 score ≥10) or “minimal depressive symptoms” (defined as having a PHQ-9 score <10) [11]. In Method 2, we categorized participants based on their baseline GAD-7 score; participants either had “anxiety symptoms” (defined as having a GAD-7 score ≥10) or “minimal anxiety symptoms” (defined as having a GAD-7 score <10) [12]. Methods 3 and 4 were based on self-reported health care utilization and treatment. In Method 3, we categorized participants as “no hospitalization history” or “history of hospitalization” based on whether they had any self-reported history of a mental health-related hospitalization in the last year. For Method 4, we categorized participants as “on medications” or “no medications” based on whether they were taking mental health–related medications at study baseline.

For the baseline behavioral data analysis, we calculated the various per-patient behavioral variables outlined in Table 1 using daily step counts and daily sleep data from activity trackers. We did not analyze weight and food logging data from apps and trackers because these data types were not commonly available in the study sample.

**Table 1:** Behavioral variables.

Variables		Definitions
**Walk metrics**		
	Daily steps	Number of steps a participant takes in a 24-hour period
	CV^a^ daily steps	Coefficient of variation of steps; measures variability of daily steps
	Steps intensity	Percent difference between median and 75th percentile day of daily steps
	Low step days	Percent of days with fewer than 500 steps walked
	High step days	Percent of days with more than 10,000 steps walked
**Sleep metrics**		
	Daily sleep	Number of hours a participant sleeps in a 24-hour period (may include naps)
	CV daily sleep	Coefficient of variation of daily sleep; measures variability of daily sleep
	Daily sleep intensity	Percent difference between median and 75th percentile day of daily sleep
	Low sleep days	Percent of days with fewer than 4 hours slept
	High sleep days	Percent of days with more than 9 hours slept

^a^CV: coefficient of variation.

### Statistical Analyses

We present the baseline demographics, health outcomes, and activity tracker–based behavioral characteristics of the participant population. Baseline sociodemographic factors, health status, and health care utilization were calculated for all enrolled participants who completed the baseline assessment. In addition, we present cross-sectional analysis based on the baseline activity tracker–based behavioral characteristics calculated for all participants who had completed the baseline assessment and used an activity tracker or fitness and health app for at least 60 of the 90 days prior to study enrollment. This threshold was set to ensure that any behavioral characteristics or patterns inferred from tracker-based behavioral data were based on a robust set of participant data rather than sparse data points. Since data collected from a variety of consumer wearable devices and apps was used in this study, no advanced preprocessing was completed. The activity tracker behavioral characteristics in this analysis focus on step and sleep data; Table 1 defines the behavioral variables examined in this analysis.

To understand how the classification methods based on validated patient reported outcomes (Methods 1 and 2) were associated with the health care utilization classification methods (Methods 3 and 4), we calculated the number of mental health–related hospitalizations and proportion of participants on mental health–related medications at baseline for each of the two groups in Methods 1 and Methods 2. We evaluated differences in the number of hospitalizations using independent *t* tests (two-tailed). We evaluated the differences in medication usage rates using a two-proportion z-test. The metrics in Table 1 were calculated for each group within each of the four classes; between-group comparisons were performed using the Mann-Whitney U test. *P* values were corrected for false discovery rates to minimize the Type 1 error rate from the multiple comparisons using the Benjamini-Hochberg procedure. All statistical tests with false discovery rate–corrected *P* values <.001 (*q* value <.05) were considered statistically significant. These results were then used to identify which behavioral variables were significantly different between groups across the four classification methods. Statistical analyses were conducted in Stata 13.1 and Python 3.5.

## Results

### Study Sample

**Figure 1:**
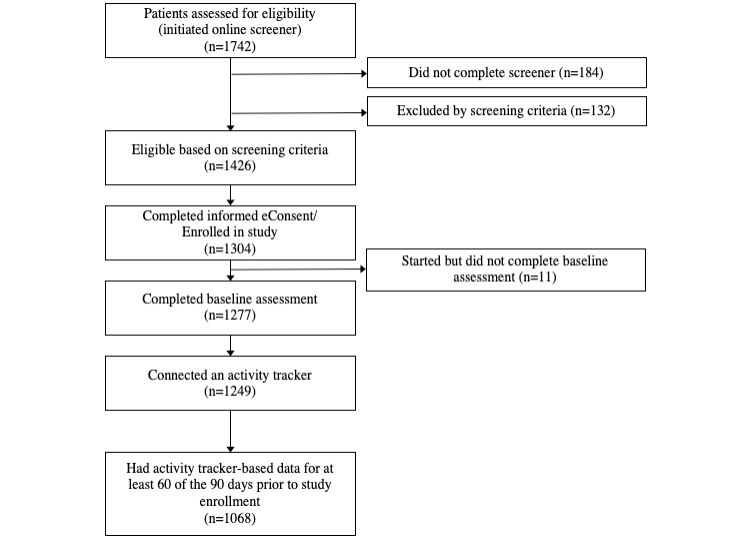
Study flow.

**Figure 2:**
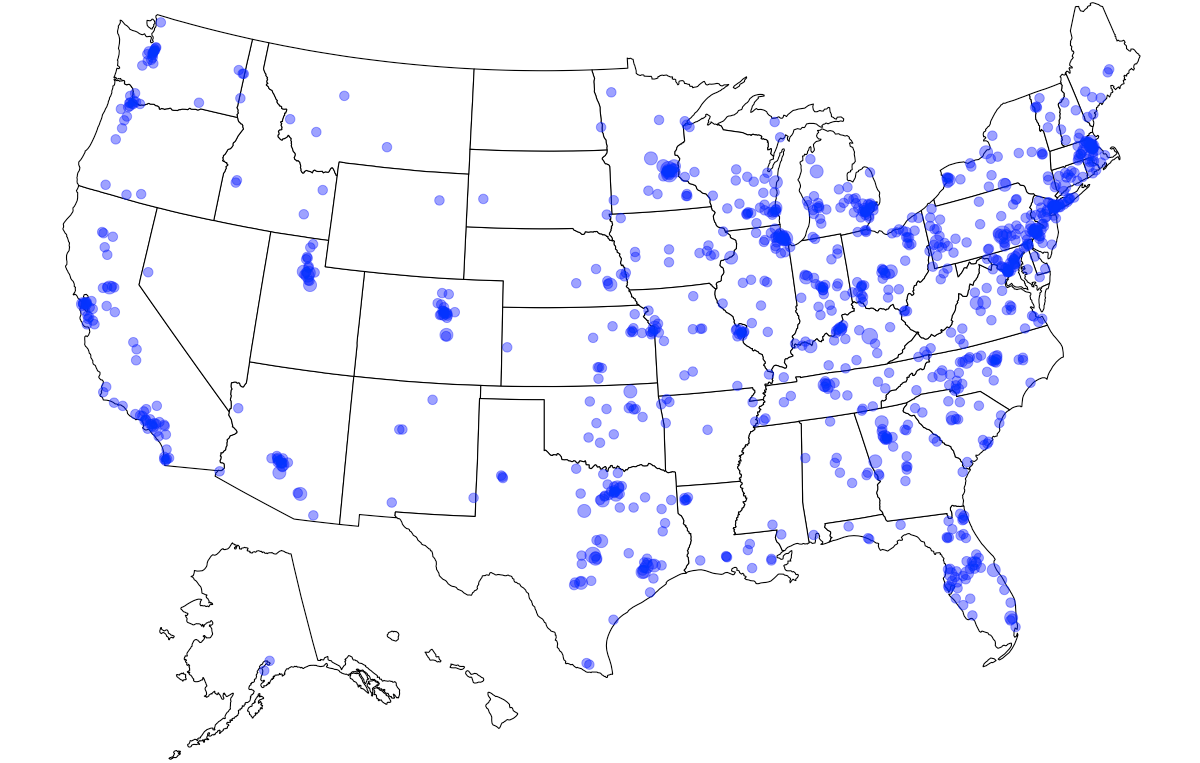
Geographic distribution of study participants. Point size is scaled by the number of participants per zip code.

Study recruitment, screening, and enrollment occurred between April 6, 2017, and May 26, 2017. A total of 1742 potential participants initiated the online screener questionnaire (Figure 1). Of those, 81.9% (1426/1742) were deemed eligible for the study. A total of 1304 individuals completed informed consent and were enrolled in the study. Of the individuals enrolled in the study, 98.8% (1288/1304) started the baseline assessment, 97.9% (1277/1304) completed the baseline assessment, 95.8% (1249/1304) connected an activity tracker to the Achievement Studies Platform, and 81.9% (1068/1304) had activity tracker–based data for at least 60 of the 90 days prior to study enrollment. Of those who completed the baseline assessment, 89.5% (1143/1277), 90.3% (1153/1277), 90.1% (1151/1277), and 89.7% (1146/1277) completed assessments at months 3, 6, 9, and 12, respectively. In total, 80.0% (1022/1277) completed all 5 quarterly assessments. Of those who completed the baseline assessment, all 50 states and Washington, DC were represented in the sample (Figure 2).

Baseline demographic, behavioral, and clinical characteristics are presented in Tables 2 and 3. The mean age of participants was 33 (SD 9) years, and the majority of the study population was female (77.2%, 994/1288) and identified as Caucasian (88.3%, 1137/1288). Participants reported on average 2.2 mental health diagnoses. The most frequently reported diagnoses were depression, with no further specification (53.3%, 687/1288); anxiety, with no further specification (47.8%, 616/1288); and generalized anxiety disorder (43.7%, 563/1288). Common comorbidities included asthma (19.7%, 252/1277), hypertension (16.1%, 206/1277), and insomnia (15.0%, 191/1277).

At baseline, 94.8% (1211/1277) of study participants reported experiencing depression or anxiety symptoms in the last year. A total of 40.4% of the study population (516/1277) had moderate to severe anxiety (GAD-7 score ≥10) and 43.7% of the study population (558/1277) had moderate to severe depression (PHQ-9 score ≥10). Approximately half of the participants were taking prescription medications for anxiety or depression and 29.2% (373/1277) were currently undergoing therapy for their anxiety or depression. Based on activity tracker data from 90 days prior to study enrollment, participants walked on average 7527 (SD 3282) steps and slept 6.92 (SD 1.63) hours in a 24-hour period.

**Table 2:** Baseline demographics and behavioral characteristics of enrolled participants.

Parameters			Values
**Demographics (n=1288)**			
	Age (years), mean (SD)		33 (9)
	**Gender, n (%)**		
		Female	994 (77)
		Male	288 (22)
		Other	6 (1)
	**Race/ethnicity, n (%)**		
		African American	22 (2)
		Asian	27 (2)
		Caucasian	1137 (88)
		Hispanic	39 (3)
		Multiracial	57 (4)
		Other	6 (1)
**Behavioral characteristics (n=1068)**			
	Daily steps, mean (SD)		7527 (3282)
	Daily sleep (hours), mean (SD)		6.92 (1.63)

**Table 3:** Clinical characteristics of enrolled participants.

Participant group and clinical characteristics			Values
**Responses from participants that started the baseline assessment (n=1288)**			
	**BMI category, n (%)**		
		Underweight (<18.5)	25 (2)
		Normal weight (18.5-24.9)	396 (31)
		Overweight (25-29.9)	371 (29)
		Obese (>30)	496 (39)
	**Mental health diagnosis, n (%)**		
		Anxiety, no further specification	616 (48)
		Bipolar disorder	93 (7)
		Depression, no further specification	687 (53)
		Generalized anxiety disorder	563 (44)
		Major depressive disorder	214 (17)
		Obsessive-compulsive disorder	109 (9)
		Panic disorder, with or without agoraphobia	156 (12)
		Phobic anxiety disorder	21 (3)
		Postpartum depression	69 (5)
		Posttraumatic stress disorder	207 (16)
		Other	113 (9)
	Number of mental health diagnoses, mean (SD)		2.21 (1)
**Responses from participants that completed the baseline assessment (n=1277)**			
	**Anxiety severity based on the Generalized Anxiety Disorder Questionnaire (GAD-7) score, n (%)**		
		Minimal or no anxiety (0-4)	302 (24)
		Mild anxiety (5-9)	459 (36)
		Moderate anxiety (10-14)	290 (23)
		Severe anxiety (15-21)	226 (18)
	**Depression severity based on the Patient Health Questionnaire (PHQ-9) score, n (%)**		
		Minimal or no depression (0-4)	272 (21)
		Mild depression (5-9)	447 (35)
		Moderate depression (10-14)	305 (24)
		Moderately severe depression (15-19)	163 (13)
		Severe depression (20-27)	90 (7)
	Symptoms of anxiety or depression in the past year, n (%)		1211 (95)
	**Common comorbidities, n (%)**		
		Asthma	252 (20)
		Hypertension	206 (16)
		Insomnia	191 (15)
		Irritable bowel syndrome	169 (13)
		Gastroesophageal reflux disease (GERD)	159 (13)
	Currently taking prescription medications for anxiety or depression, n (%)		673 (53)
	**Types of therapies utilized for anxiety or depression, n (%)**		
		One-on-one psychotherapy or counseling, in-person	308 (24)
		Psychotherapy or counseling through a mobile or online app	11 (1)
		Support group, in-person	29 (2)
		Support group through a mobile or online app	23 (2)
		Other	40 (3)
		None of the above	904 (71)
	Has a primary care provider, n (%)		1072 (84)
	Number of doctor’s office visits per year (for any reason), mean (SD)		3.46 (7.26)
	Number of urgent care clinic visits per year (for any reason), mean (SD)		0.85 (1.28)
	Number of emergency room visits per year (for any reason), mean (SD)		0.43 (1.19)
	Number of emergency room visits per year (for anxiety or depression), mean (SD)		0.07 (0.60)
	Had anxiety- or depression-related emergency room visit in past year, n (%)		50 (4)
	Number of hospitalizations per year (for any reason), mean (SD)		0.13 (0.48)
	Number of hospitalizations per year (for anxiety or depression), mean (SD)		0.02 (0.17)
	Had anxiety- or depression-related hospitalization in past year, n (%)		19 (1)

### Disease Severity, Health Care Utilization, and Medication Use

Baseline health care utilization for subgroups of the study population based on the disease severity classification methods (Methods 1 and 2) is presented in Table 4. Participants with depressive symptoms (based on PHQ-9) reported on average 0.011 more mental health–related hospitalizations in the past year than participants with minimal depressive symptoms, although this was not statistically significant (0.025 versus 0.014; *t*_1275_=–1.16, *P*=.25). Similarly, participants with anxiety symptoms (based on GAD-7) reported 0.003 more mental health–related hospitalizations in the past year than participants with minimal depressive symptoms, although this was not statistically significant (0.021 versus 0.017; *t*_1275_=–0.43, *P*=.67).

Participants with depressive symptoms were significantly more likely to be taking a mental health–related medication at baseline (57.9% versus 48.7%; *z*=–3.27, *P*=.001, two-tailed). A higher proportion of individuals with anxiety symptoms were taking mental health–related medications than those with minimal anxiety symptoms; however, this difference did not reach statistical significance (54.8% versus 51.3%; *z*=–1.26, *P*=.21, two-tailed).

**Table 4:** Health care utilization, medication usage, and disease severity.

Parameters	Method 1		Method 2	
	Minimal depressive symptoms (n=719)	Depressive symptoms (n=558)	Minimal anxiety symptoms (n=761)	Anxiety symptoms (n=516)
Mental health–related hospitalizations in past year, mean (SD)	0.014 (0.16)	0.025 (0.19)	0.017 (0.19)	0.021 (0.14)
Taking mental health–related medication at study baseline, n (%)	350 (48.7)^a^	323 (57.9)^a^	390 (51.3)	283 (54.8)

^a^*P*=.001.

### Behavioral Analysis

The baseline behavioral metrics for subgroups of the study population based on the four classification methods is presented in Tables 5 and 6. Individuals with depressive symptoms were less active than those with minimal depressive symptoms. On average, individuals with depressive symptoms took 603 fewer steps per day than those with minimal depressive symptoms (*u*=155751.0, *P*<.001) and had 4.3% fewer high step days than those with minimal depressive symptoms (*u*=154478.0, *P*<.001). Individuals taking depression- or anxiety-related medication at baseline were also less active than those who were not taking medication. On average, individuals taking medication took 429 fewer steps per day than those who were not taking medication (*u*=127449.0, *P*<.001). Individuals with depressive symptoms or taking depression- or anxiety-related medication had more inconsistent sleep patterns, as measured by a higher coefficient of variation (CV) for daily sleep and higher daily sleep intensity, compared to those with minimal depressive symptoms or not on treatment. Individuals with a history of depression- or anxiety-related hospitalization also had more inconsistent sleep patterns, as measured by a higher CV for daily sleep, than those who had not been hospitalized. Comparing individuals based on anxiety symptoms (Method 2) did not yield any statistically significant differences between the two subgroups.

**Table 5:** Behavioral variables by classification method (Methods 1 and 2).

Variables		Method 1		Method 2	
		Minimal depressive symptoms	Depressive symptoms	Minimal anxiety symptoms	Anxiety symptoms
**Walk metrics**					
	Participants, n	617	451	646	422
	Daily steps, mean (SD)	7782 (3759)	7179 (3882)^a^	7684 (3692)	7288 (4004)
	CV^b^ daily steps	0.48	0.50	0.48	0.50
	Steps intensity, %	35.2	41.1	36.6	39.4
	Low step days, %	2.1	2.8	2.3	2.6
	High step days, %	28.5	24.2^a^	27.7	25.1
**Sleep metrics**					
	Participants, n	501	373	526	348
	Daily sleep, mean (SD)	6.97 (1.61)	6.86 (1.60)	6.93 (1.60)	6.91 (1.69)
	CV daily sleep	0.23	0.26^a^	0.24	0.25
	Daily sleep intensity, %	14.4	16.4^a^	15.5	14.8
	Low sleep days, %	4.1	5.4^a^	4.4	5.1
	High sleep days, %	8.3	9.3	8.2	9.6

^a^*P*<.001, false discovery rate–corrected *P* value for multiple comparisons.

^b^CV: coefficient of variation.

**Table 6:** Behavioral variables by classification method (Methods 3 and 4).

Variables		Method 3		Method 4	
		No history of hospitalization	History of hospitalization	Not on treatment	On treatment
**Walk metrics**					
	Participants, n	1055	13	506	562
	Daily steps, mean (SD)	7515 (3788)	8540 (6118)	7753 (3386)	7324 (4168)^a^
	CV^b^ daily steps	0.49	0.48	0.47	0.50
	Steps intensity, %	37.6	45.7	36.9	38.4
	Low step days, %	2.4	2.1	2.0	2.7
	High step days, %	26.7	29.3	28.6	25.0
**Sleep metrics**					
	Participants, n	863	11	406	468
	Daily sleep, mean (SD)	6.93 (1.63)	6.59 (1.71)	6.88 (1.55)	6.96 (1.70)
	CV daily sleep	0.25	0.36^a^	0.23	0.26^a^
	Daily sleep intensity, %	15.2	18.0	13.4	16.9^a^
	Low sleep days, %	4.6	10.4	4.4	4.9
	High sleep days, %	8.7	12.0	7.7	9.7

^a^*P*<.001, false discovery rate–corrected *P* value for multiple comparisons.

^b^CV: coefficient of variation.

## Discussion

### Principal Findings

This virtual 1-year observational study was designed to create a longitudinal data set of validated patient reported outcomes; health care utilization and treatment; and behavioral data for individuals with anxiety or depression to understand how passively collected behavioral data can help provide insights into an individual’s overall health.

This analysis found that certain passively tracked behavioral traits are associated with more severe forms of anxiety or depression, as indicated by validated disease severity scales, health care utilization, and medication usage. In particular, severe forms of depression, as measured by PHQ-9, were significantly associated with inconsistent sleep patterns and more disordered sleep. Individuals who were taking anxiety- or depression-related medications slept directionally more but also had more inconsistent sleep patterns than individuals not on medication. Inconsistent sleep patterns were also a trait associated with individuals who had been hospitalized for their anxiety or depression. Further analyses should be conducted to understand whether sleep outcomes are associated with medication use (eg, side effects) or with the severity of the disease.

Our findings on associations between mental health severity and health care utilization were mixed. The baseline analysis found no significant difference in self-reported history of mental health–related hospitalizations among individuals with different levels of anxiety and depression severity, as measured by GAD-7 and PHQ-9, respectively. Given that the frequency of mental health hospitalizations per year was relatively low, we may not have had a large enough sample size to show statistical significance. Furthermore, while we found that a larger proportion of individuals with depressive symptoms were on medication at baseline than individuals with minimal depressive symptoms, other studies have found a trend of increasing medication usage in people with minimal depressive symptoms [13,14]. The proportion of individuals with anxiety symptoms who were on medication at baseline was directionally more than the proportion of individuals with minimal anxiety who were on medication at baseline, although this was not statistically significant. One potential explanation of this finding is that those with anxiety use medication as treatment less often than those with depression as nonpharmaceutical alternatives are effective (eg, cognitive behavioral therapy, meditation, exercise) [15-18]. Further analyses of the collected longitudinal data may be useful for examining the association between changes in disease severity over time and participants starting and stopping medications over time.

### Strengths and Limitations

The design of this study has strengths as well as some limitations. This study was conducted completely virtually, which allowed for a geographically diverse population to enroll and participate in the study; this may increase the overall generalizability of the analyses and findings to a broader US population. Furthermore, about 90% of participants completed the online questionnaires at months 3, 6, 9, and 12, suggesting that it is feasible to engage large, digital populations in virtual studies to better understand health behaviors and outcomes. All behavioral data were passively collected via activity trackers and fitness and health apps, allowing us to capture everyday health behaviors (eg, step count) that may not otherwise be captured in traditional health care settings (eg, routine visits to a doctor’s office). Participants could complete their online assessments in the privacy of their own homes with minimal effort and disruption; they did not have to travel to clinics or have any in-person interactions when providing study data. This allowed us to capture truly real-world data; we were able to analyze this data combined with self-reported health outcomes to better understand the impact of anxiety and depression on individuals’ daily lives and behaviors. This suggests that passively collected digital data can help us characterize mental health illnesses like anxiety and depression beyond what is already captured in clinical measures (eg, GAD-7 and PHQ-9). Another strength is the 1-year duration of the study, which will allow us to examine how changes in behavioral data are associated with changes in disease severity (ie, worsening and improvement of symptoms) over time.

One of the limitations of this study is that it lacked a healthy comparison control group, which may limit findings in future analyses. However, as a first step to understand the associations between passively collected behavioral data and health outcomes, the observational nature of this study is likely sufficient. Although the study population was geographically diverse, the majority of participants were young, female, and Caucasian. However, our results are consistent with the epidemiology of anxiety and depression in the general population, showing a higher prevalence of anxiety and depression among younger age groups, women, and people who identify as White [19-22].

### Conclusions

These promising initial findings suggest that longitudinal behavioral and health outcomes data may be useful for developing digital measures of health for mental health symptom severity and progression. In future analyses, we will aim to understand how longitudinal changes in behavioral data can be mapped to changes in disease severity and health care utilization over a 12-month period.

## Abbreviations

CV: coefficient of variation
GAD-7: Generalized Anxiety Disorder Questionnaire
PHQ-9: Patient Health Questionnaire
